# Laser Discoloration in Acrylic Painting of Visual Art: Experiment and Modeling

**DOI:** 10.3390/ma14082009

**Published:** 2021-04-16

**Authors:** Khairul Fikri Tamrin, Kaveh Moghadasi, Marzie Hatef Jalil, Nadeem Ahmed Sheikh, Shahrol Mohamaddan

**Affiliations:** 1 Department of Mechanical and Manufacturing Engineering, Faculty of Engineering, Universiti Malaysia Sarawak (UNIMAS), Kota Samarahan 94300, Sarawak, Malaysia; kave.moghaddasi@gmail.com; 2 Department of Mechanical Engineering, Faculty of Engineering, University of Malaya, Kuala Lumpur 50603, Malaysia; 3 Department of Design Technology, Faculty of Applied and Creative Arts, Universiti Malaysia Sarawak (UNIMAS), Kota Samarahan 94300, Sarawak, Malaysia; marzie.hatef@gmail.com; 4 Department of Mechanical Engineering, Faculty of Engineering & Technology, International Islamic University, Islamabad 44000, Pakistan; ndahmed@gmail.com; 5 Department of Bioscience and Engineering, Shibaura Institute of Technology, College of System Engineering and Science, Fukasaku 307, Minuma-ku, Saitama 337-8570, Japan; mshahrol@shibaura-it.ac.jp

**Keywords:** laser, processing, machining, art, painting, material, modeling

## Abstract

This study discloses a method for painting artwork using a CO_2_ laser. The continuous-wave laser beam, at a predetermined heat flux and a predetermined number of laser beam passes, mixes and displaces the plurality of colored polymer-based compositions, respectively, by way of melting and vaporizing them. Experiments showed a great accuracy of colors and designed patterns between the computer aided design (CAD) drawing and what was achieved after laser discoloration. It was found that lower values of power and speed provide sufficient energy and time to make a melt pool of colors and cause their vaporization from the surface. A detailed numerical simulation was performed to obtain a detailed understanding of the physics of laser interaction with paint using ABAQUS software. The comparative analysis indicated that the top layer of paint (including yellow and green colors) melted upon increasing cutting speed and employing one laser pass. For blue and red paints, two passes of lasers are required; in the case of red color, lower laser speed is also necessary to intensify the heat. This method can be applied for making art designs on each surface color because it is based on melting and vaporization using a laser.

## 1. Introduction

Visual arts include fine art, decorative art, and contemporary art, with a primary focus on creating pieces of compelling work that could convey messages of emotion, ideas, or information. Historically, every generation of every nation has contributed to the development of various virtual art forms. Today, modern technologies enable humanity to broaden ways of expression, allowing materials and media to interact in extraordinary ways [1].

Laser is often thought of as an industrial tool, although its applications are myriad. Laser machining has become a crucial manufacturing tool for the production of numerous manufactured parts and components with complex geometrical designs. It is widely accepted over conventional methods and is currently employed in the biomedical, automotive, aerospace, and defense sectors for reasons of precision, flexibility, and accuracy. The laser beam interacts with the electrons of the material, and part of the energy is absorbed, producing a highly localized rise in temperature up to melting, vaporization, or chemical state change. These different physical phenomena that govern the laser–material interaction mainly depend on chemical and physical properties of the material such as absorptivity and thermal conductivity, as well as the laser characteristics such as wavelength and power density.

For similar reasons, lasers have been applied for etching and engraving of visual arts (e.g., portrait and scenery) on wood-based surfaces, limited to grayscale images only. With the assistance of high-power lasers, laser coloration of metals is now possible. In principle, the change of optical properties, e.g., the reflectance of the stainless steel [2,3] and titanium [4,5] surfaces, in the visible range occurs due to thin oxide film formation when exposed to laser radiation [6,7]. There is also another mechanism of laser coloration. It is based on the diffraction of light on periodic surface structures [8] that are produced because of interference between laser radiation and an electromagnetic wave surface [5,9,10]. This method allows altering color depending on the viewing angle.

Acrylic, watercolors, oil, and pastel colors are the four classical means of artistic expression in painting. Mastering brush techniques plays a pivotal role in delivering precise expression, but this comes with years of practice, which in turn creates a stumbling block for enticing a new generation to learn and love arts. In this research, we propose a new technique of acrylic painting in visual art from the point of view of modern photonics and laser technology. Laser discoloration of acrylic paints could provide a broad palette of colors and the ability to control the result with very high accuracy to afford an almost unlimited potential for creative work. Therefore, one can consider a laser as a brush in visual art. To the best of the authors’ knowledge, there is no published article or artwork concerning the application of laser for discoloration of acrylic paints. Here, we aim to multilaterally demonstrate that lasers can be used not only as highly useful industrial tools but also as a new exciting means for visual art. Laser–acrylic interaction is inherently complex, underpinned by the complex interaction between various laser processing parameters (laser power, speed, standoff distance), acrylic properties (absorptivity, melting and boiling temperatures), and art preparation. The process involves four main mechanisms, namely melt shearing or fusion, vaporization, resolidification, and chemical degradation [11]. Generally, one of these mechanisms is dominant over the rest for a particular type of acrylic color. For instance, an initial coupling of a laser beam with an acrylic surface is governed by the material’s absorptivity and surface roughness and also depends on the laser wavelength [12]. The methodology is described in the following section.

## 2. Materials and Methods

### 2.1. Materials

**Figure 1:**
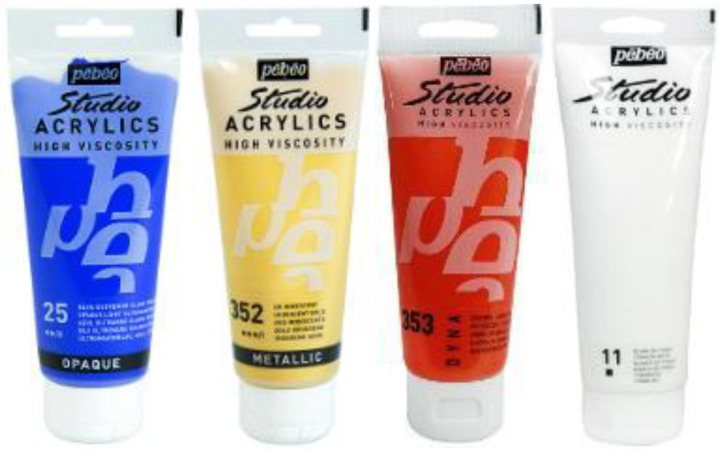
Acrylic paints used in the experiment (brand: Pebeo, Gémenos, France).

**Figure 2:**
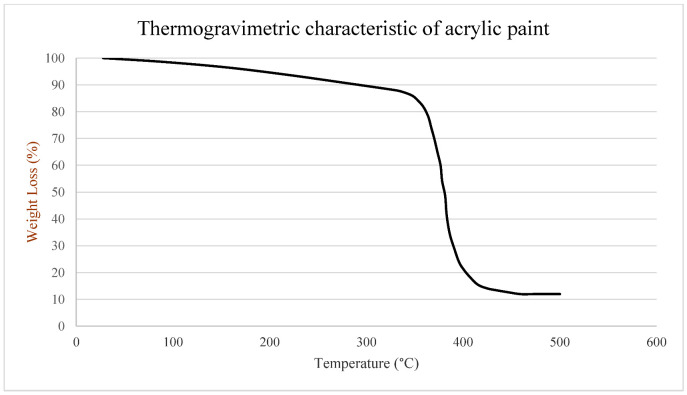
Thermogravimetric analysis (TGA) result of acrylic paint [13].

Acrylic paints are made using the highest quality acrylic resin to produce acrylic polymer emulsion colors and mediums. All colors contain pure pigments in a 100% acrylic polymer emulsion. The finest quality paints offer the purest color and provide the artist with all the essentials for creative and artistic success. Acrylics are ideal for contemporary and experimental applications. The colors dry very rapidly (remaining workable for 10–40 min), making them well-suited for applications that require masking, rapid layering, and textural application. Figure 1 shows acrylic paints used for the experiments consisting of white and three primary colors (red, blue, and yellow) for painting. A 25 mm brush was used to apply the paints. Figure 2 shows thermogravimetric characteristics of acrylic paint where its melting point starts at 300 °C and its vaporization temperature occurs at 400 °C in which it loses 90% of its weight. Therefore, it is essential to select appropriate laser speed and power for melting (mixing colors with each other) and vaporizing (eliminating color from the surface).

Acrylics can be utilized on almost any surface (e.g., paper, canvas, brick, wood). Canvas is one of the most popular art supplies for painting with acrylics. There are two types of canvas: linen and cotton. Linen is considered superior because it is smoother, stiffer, and stronger than cotton. Linen canvas is flexible and is therefore appropriate to stretch. In general, artists and galleries prefer paintings on canvas that have the staples in the back, rather than on the side. Here, a pre-stretched linen canvas was bought and used to perform the experiments.

### 2.2. Methods

#### 2.2.1. Laser Equipment

**Figure 3:**
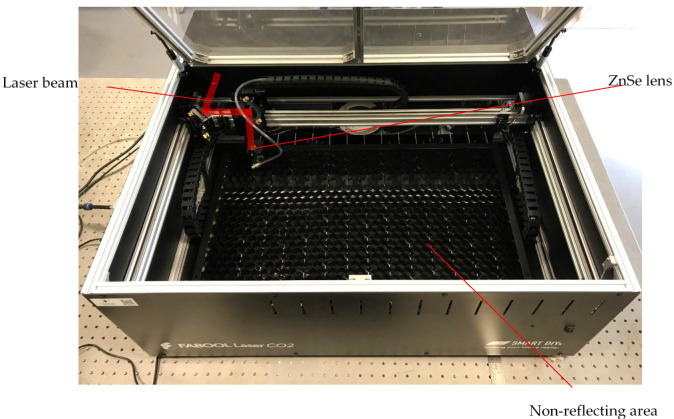
CO_2_ laser machine.

**Figure 4:**
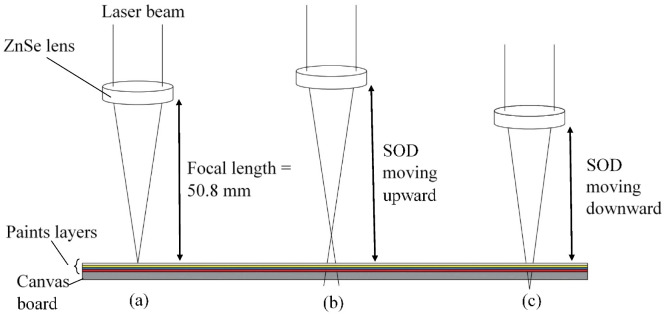
Schematic diagram of focal plane position and standoff distance relative to the surface of the workpiece, (**a**) ZnSe lens at focal length, (**b**) ZnSe lens at upper position, (**c**) ZnSe lens at lower position.

A 40 W continuous-wave CO_2_ laser (FABOOL CO_2_ laser, smartDIYs, Minami-Alps, Japan) as shown in Figure 3, was used to perform the experiment. The wavelength and beam spot size of the laser beam are 10.6 µm and 200 µm, respectively. With a working area of 600 mm × 440 mm, the maximum speed limit is 133.3 mm/s. The position of the ZnSe focal lens relative to the top surface of the material is defined as stand-off distance (SOD), as shown in Figure 4. At SOD of 50.8 mm, the beam spot is exactly on the surface of dried paints of the workpiece, which is similar to the focal length of the lens. The affected area can be adjusted by moving the focal lens upward or downward. The laser parameters can be digitally manipulated through a built-in FABOOL software, allowing control of multiple variable parameters for graphics handling.

#### 2.2.2. CAD Technology

**Figure 5:**
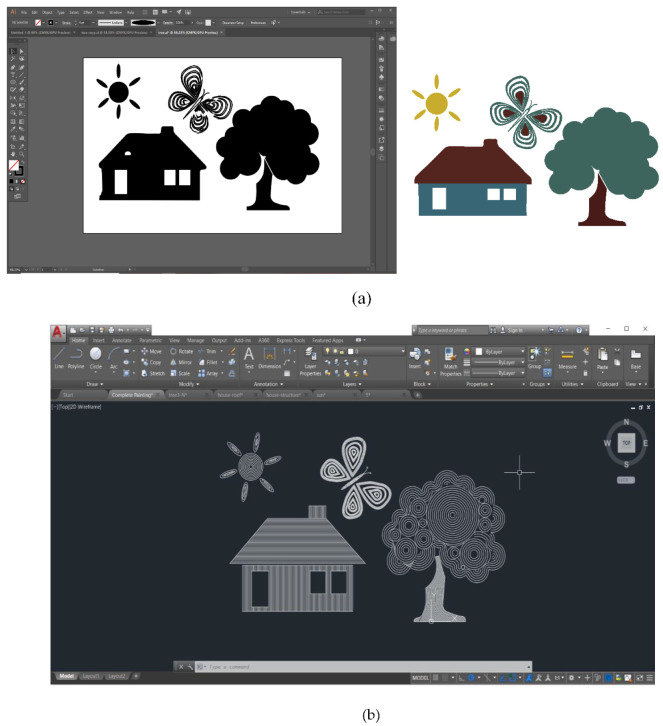
(**a**) First illustration of drawing in Adobe Illustrator. (**b**) Imported drawing (.dxf file) in AutoCAD to export file for laser software.

CAD technology was used to combine digital design with digital laser processing systems. Original artwork was developed into design work using creative CAD software: Adobe Illustrator (version 2017, Adobe Inc., San Jose, CA, USA) and AutoCAD (version 2018, Autodesk). Design files were exported and converted into an appropriate format (.dxf) for FABOOL laser software. Figure 5 shows the procedure of making the final drawing that results in laser head movement to create the painting on the top surface of the canvas.

A digital microscope (Model SME-F8BH, AmScope, Irvine, CA, USA) was used to capture micrographs of the painted surface. A positive USAF 1951 test target was used for the calibration of the microscope. After calibration, it was found that 1 pixel is equal to 0.547 μm for 4× magnification objective. The microscope was equipped with a Complementary metal–oxide–semiconductor(CMOS) camera with a resolution of 5 MP.

## 3. Experimental Procedures

**Figure 6:**
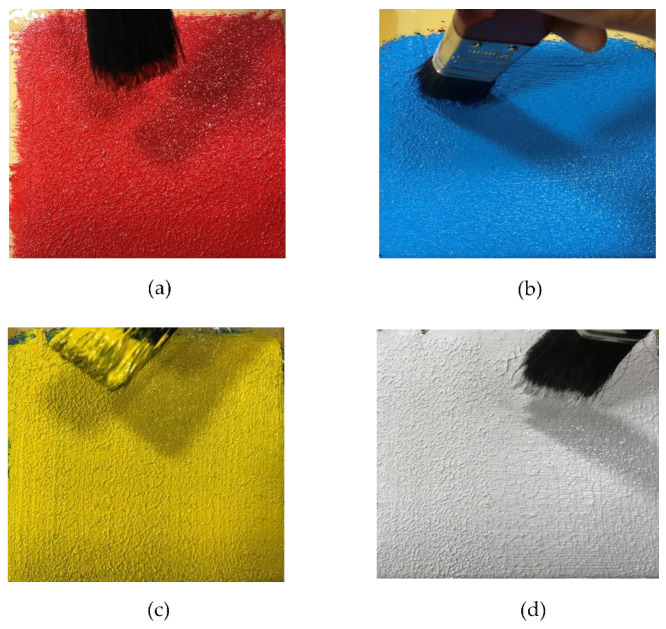
Four steps of applying paints: (**a**) Step 1 (red color), (**b**) Step 2 (blue color), (**c**) Step 3 (yellow color), and (**d**) Step 4 (white color).

Firstly, all colors were individually applied on the surface of the canvas board using a 25 mm brush. The layers in the sequence from bottom to top were red, blue, yellow, and white (brand: Pebeo, Gémenos, France), as shown in Figure 6. It is noted that different brushing techniques would result in differences in surface roughness and could be desirable for enhancing absorptivity of the laser beam on the acrylic surface.

Three finished surfaces with different surface roughness values were produced to be analyzed. Among these three, one is more appropriate to provide the desired result by absorbing laser energy. The surface roughness measurement was done using surface roughness tester (Model: SRT-6200, BESTONE, Shanghai, China). It was found that the surface with lower roughness results in better output colors as the absorbed laser energy is not overly reflected by the rugged surface, and it causes smoother melting/vaporizing of paints, while for higher roughness, burning colors and failure to achieve the true color were observed, as shown in Table 1.

### Preliminary Experiments

To achieve each color using CO_2_ laser, some preliminary experiments were conducted at different laser powers, speeds, and Stand-off distances (SODs). By increase or decrease in SOD, the size of the beam spot would vary proportionally (Figure 4). The change of SOD away from the focal position leads to a larger heat-affected area and creates a melt pool of colors to produce a true combination of them. Although an increase in laser power at lower speed induces more energy on the surface of the color and it was observed that the color was completely burnt, a decrease in laser power provides optimum energy to acquire the appropriate color by laser discoloration. For this reason, different laser powers and speeds at different SODs were selected, for which some preliminary results are shown in Table 2.

**Figure 7:**
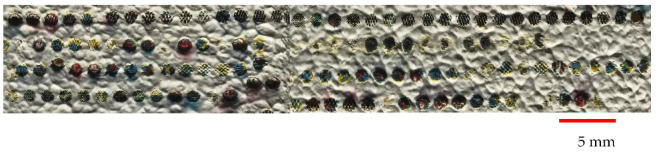
Preliminary experiments conducted by applying smaller circle size and the following ranges of laser input parameters: 1 W ≤ P (laser power) ≤ 30 W, 1 mm/s ≤ V (laser speed) ≤85 mm/s.

The following figures show the complete set of preliminary experiments performed to acquire desired results and discover appropriate laser parameters. Firstly, it can be seen in Figure 7 that experiments were started using a smaller circle size. Owing to this, the laser was not able to produce the melt pool, and the final results were not acceptable.

When switching the CAD drawing to a larger circle size, it can be observed that the influence of laser parameters is more obvious than that in the preceding experiments; more importantly, by altering the parameters and selecting the optimal parameters, appropriate colors were obtained. Table 3 shows different sets of experimental results and the ranges of laser parameters for each set.

## 4. Result and Discussion

The preliminary results showed that more acceptable results are achieved by positioning the focal lens at an upper location and selecting the lowest laser power and minimum speed. It is interesting to note that although SOD at a lower position produces an extensive affected area, it cannot produce a suitable result. This can be attributed to the nature of the Gaussian beam in which the power density is not evenly distributed across the beam spot [14,15]. Table 2 shows samples at SODs of 47.8 and 48.8 mm, both of which result in a burnt area at the top surface of the paints. As can be observed, the increment in cutting speed enhances the cooling effect of the paint layers and reduces the temperature in the inner layers. It leads to less penetration of the laser beam and a reduction in the occurrence of a melt pool of paints [16,17]. This results in the vaporization of the paints were vaporized and samples appearing as a carving of paints on the surface. On the contrary, with the increase in laser power, the material absorbs more thermal energy emitting from the incident laser beam, which leads to more evaporation and burning of the paints [18,19]. Thus, it is essential to select low power at low speed to make a melt pool of colors and mix them to provide a different spectrum of colors. For instance, green color, which is a combination of yellow and blue colors, could be produced by positioning SOD at 52 mm and selecting laser power of 0.4 W and speed of 5 mm/s. To produce blue color or red-brown color, it is necessary to use double passes. The reason is that in the first pass, when appropriate laser parameters are selected, the laser starts mixing yellow and top layer of blue paints. A laser beam at lower laser power and optimum laser speed cannot deeply penetrate into layers of paints and only involves the top layers; therefore, a mixture resulting in green color illustration is produced. If the blue color is needed, the laser should be set at higher laser speed at the same power to act as a carving machine to eradicate the top mixed layer. On the contrary, to obtain a red-brown color, it is important to reduce the speed, which provides enough time for the laser beam to penetrate and melt the paints to obtain the desired color.

**Figure 8:**
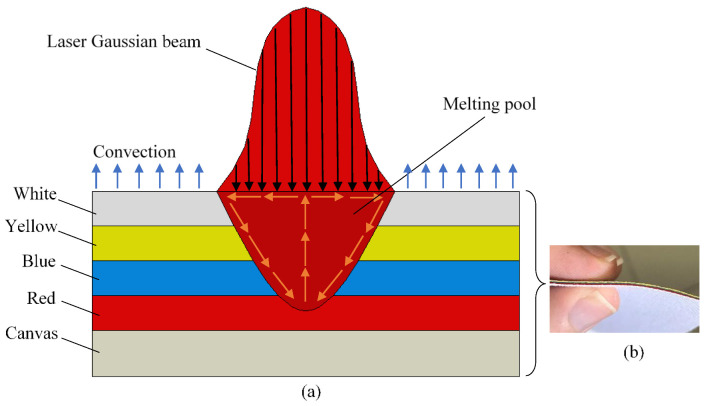
(**a**) Schematic of laser melting and the heat transfer in the molten pool. (**b**) The applied layers of paints on top of the canvas.

**Figure 9:**
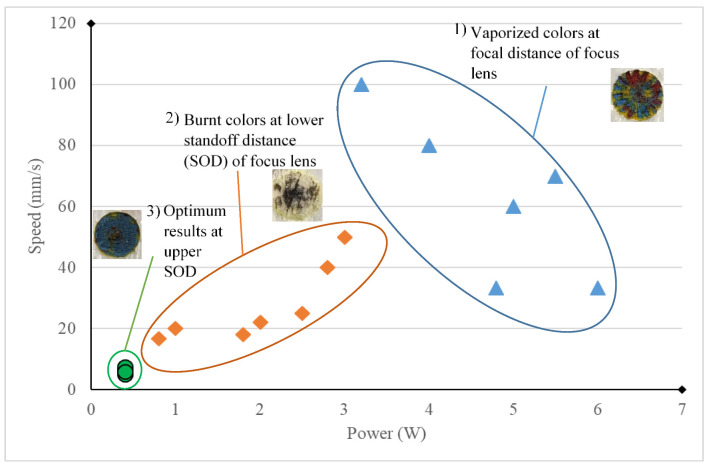
Laser process parameters in three different zones based on laser power, speed, and stand-off distance (SOD).

Figure 8 displays the schematic diagram of the laser melting physical model. The laser beam with a Gaussian power distribution works as a heat source. A series of physical phenomena occur in an essential operation of laser decoloration: absorption and scattering of laser radiation, heat transfer, color melting, fluid flow within the molten pool, and solidification. Additionally, as shown in Figure 9, the difference is clearly visible if some data are plotted, corresponding to three combinations of process conditions:

1. High power, high speed at focal length (50.8 mm).
2. Medium power, medium speed at lower standoff distance (47–49.8 mm).
3. Low power, low speed at upper standoff distance (52 mm).

However, in the first and second cases, the area was burnt or vaporized unevenly, and the melt pool was not properly created. The analysis allows the conclusion that, for the laser utilized, the increment in laser speed enhances the cooling effect of the paint layers and reduces the temperature in inner layers, resulting in less penetration of laser beam and reduction in the occurrence of the melt pool of paints. On the contrary, the increase in laser power enhances the irradiated energy, which leads to the sudden vaporization of color from the surface and the production of a region with a variety of colors, as can be seen in region 1 of Figure 9. Changing the standoff distance and positioning the lens closer to the surface of the specimen creates a burnt region of colors (region 2, Figure 9). The optimum parameters were discovered at higher standoff distance and lower laser power and speed, which gives enough time to make the melt pool of colors and prevents the induction of great amounts of energy and the burning of the colors (region 3, Figure 9).

**Figure 10:**
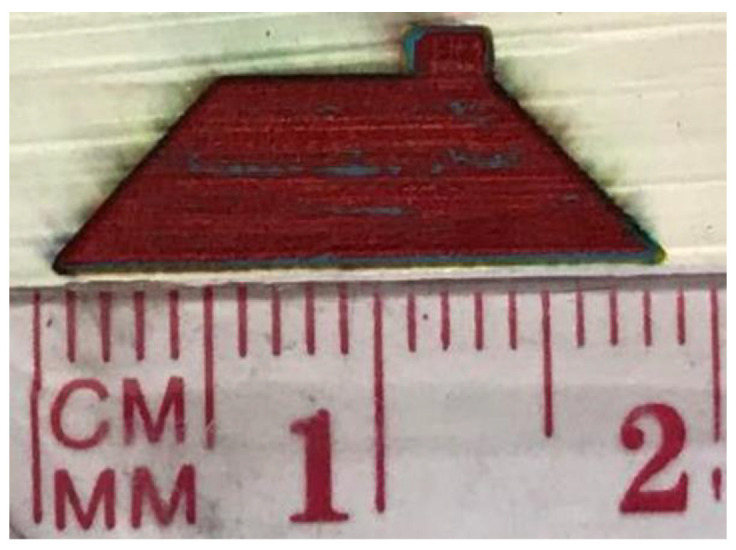
Roof part of the painting in red color.

To achieve the final painting result, the colored surface was decolorized by CO_2_ laser until it reached the exact color of each section of the painting. For instance, red was selected as the color for the roof. Therefore, the laser was adjusted to create red colors based on input parameters of laser power and speed. Figure 10 shows the roof with red color. Based on CAD design, the laser head moved in a horizontal direction to melt the color and first began vaporizing the yellow layer and part of the blue layer. Thereafter, for the next pass, it began to melt and vaporize the blue layer to reach the red color.

**Figure 11:**
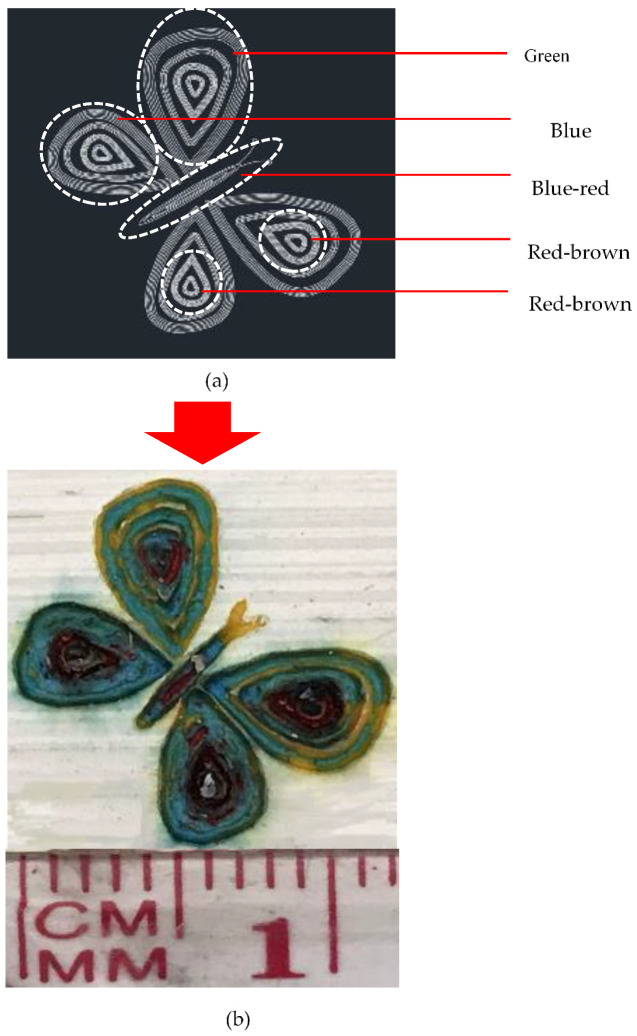
(**a**) CAD design of butterfly and definition of each part’s color. (**b**) Final output of laser painting.

To make a colorful butterfly previously created in AutoCAD, different parameters were set in the laser software. The procedure is shown in Figure 11. It can be observed that each wing has its specific colors based on the defined pattern in the CAD design (Figure 11a). These patterns were entered into the laser software separately to decolorize the painted layer by laser using different parameters. The final painting is illustrated in Figure 11b.

**Figure 12:**
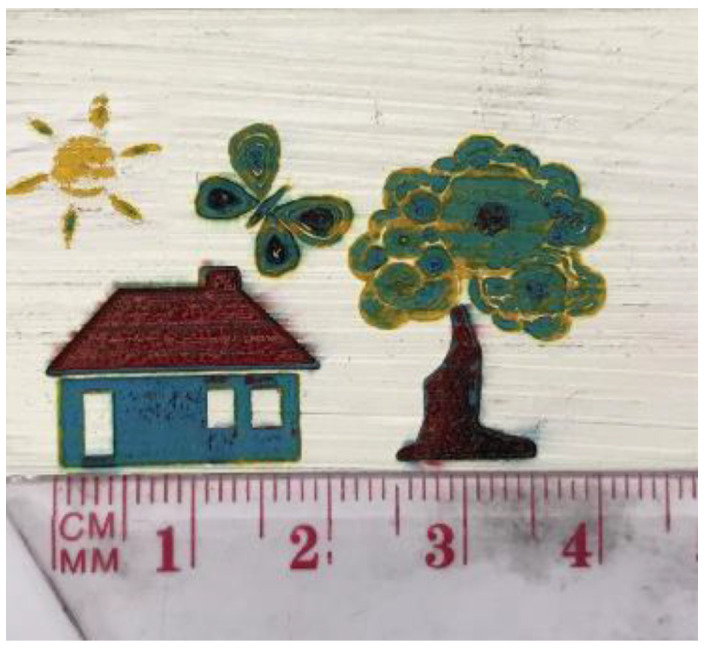
Final painting.

It is interesting to note that using a laser for painting has some unique advantages such as noncontact process, automation adaptability using software, and great accuracy in creating shapes in the millimeter scale. The final painting is shown in Figure 12. The melted regions of colors show how the laser can precisely provide a melt pool of colors and, by moving exactly in defined directions, can produce a painting in the millimeter scale. Of note, the specified time to create this painting using a laser was less than 5 min. However, if the size of the painting is increased, the laser beam would need to traverse a larger area, and it would take longer to finish the whole painting process. Therefore, the laser is an agile technology that has the potential to be used for combining processing stages and producing the final result rapidly.

**Figure 13:**
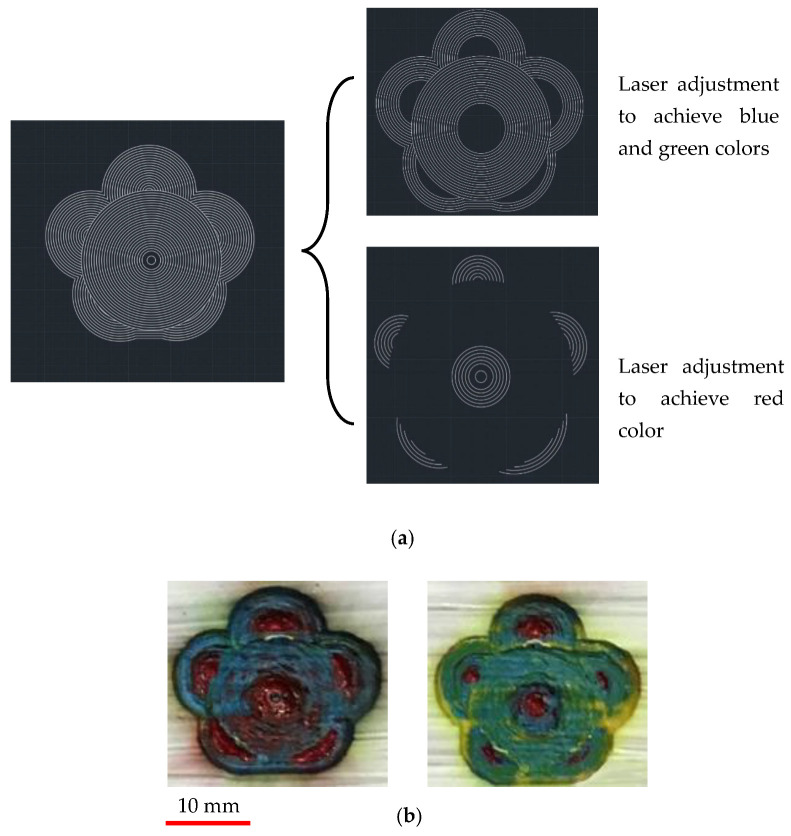
(**a**) Preparation of the CAD design to achieve different colors for each section of the flower. (**b**) Final result of colory flowers.

For more investigation, a picture of a flower was imported and modified in AutoCAD and then exported to laser software. Figure 13a shows the stages of making CAD files to import separately in Fabool software to acquire the desired colors. Two different results of painting based on different laser parameters were obtained, as shown in Figure 13b. One sample is a combination of red-brown and blue colors, while the other sample is a combination of red and green colors.

**Figure 14:**
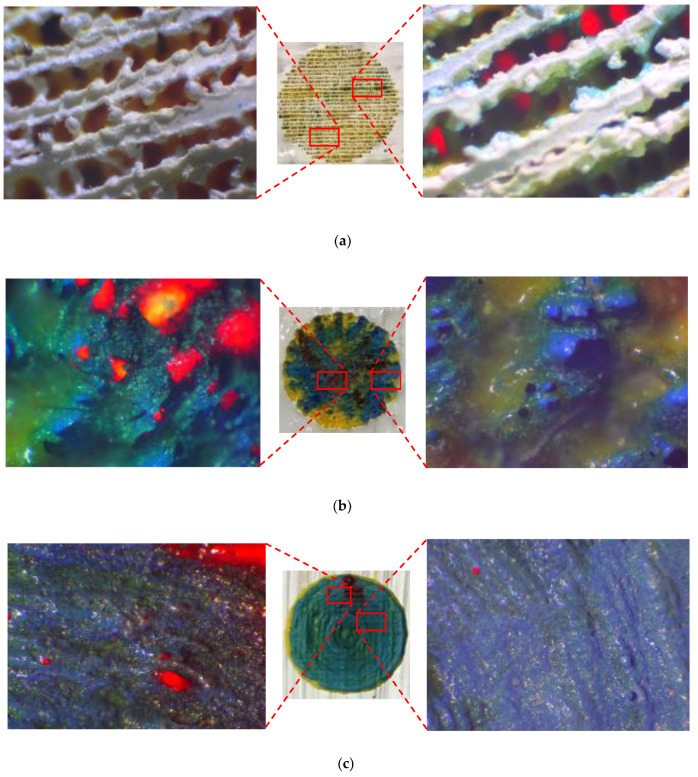
Micrographs of preliminary experiments on colored surface with different roughness (Ra) measurements: (**a**) Ra = 10.13, (**b**) Ra = 8.6, and (**c**) Ra = 5.95.

It can be seen in Figure 14 that each surface finish of colors due to having different surface roughness provides different intensity for each color. It has to be noted that lower surface roughness (Figure 14c) leads to greater color intensity as the absorbed laser energy is not overly reflected by the rugged surface, and it causes better melting/vaporizing of paints, while it appears that the absorbed energy contributes more to residual heat and burning of colors when effective surface area increases (Figure 14a,b). This can be attributed to the many reflections occurring after laser irradiation on the rugged colored surface, whereby the laser beam is not intensively focused and the melt pool is not made properly.

**Figure 15:**
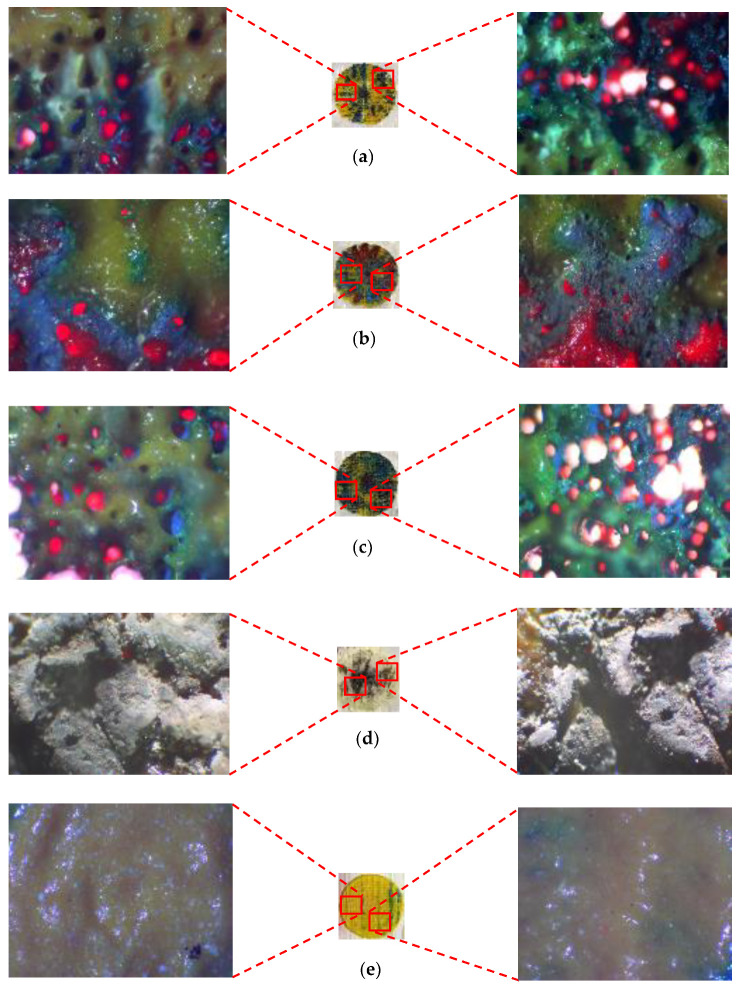
Results for different laser parameters: (**a**) P = 3.2 W, V = 100 mm/s, SOD = 50.8 mm; (**b**) P = 2 W, V = 50 mm/s, SOD = 50.8 mm; (**c**) P = 4.8 W, V = 33.33 mm/s, SOD = 50.8 mm; (**d**) P = 0.8 W, V = 16.66 mm/s, SOD = 47.8 mm; and (**e**) P = 0.4 W, V = 6.66 mm/s, SOD = 52 mm.

After finding the appropriate surface roughness to execute laser decoloration, different laser parameters were assessed in terms of their ability to achieve the true color needed for the output CAD drawing. For the result of each set of parameters assessed, micrographs were extracted. Figure 15 shows the results for different laser parameters. It can be seen in Figure 15a–c that when the laser head is set to a focal length equal to 50.8 mm, the focused laser beam forms a high-energy column of heat that enters the paint layers and results in vaporization and burning of polymers. By moving the laser head upward or downward, the size of the beam spot would vary proportionally, resulting in a larger heat-affected area and creating a melt pool of colors to produce a true combination of them (see Figure 15e). It can be observed that when the laser power dwindles at low scanning speed, optimum energy is provided to create the appropriate color by laser discoloration. It is interesting to note that although SOD at a lower position produces an extensive affected area as well, it cannot produce a suitable result. This can be attributed to the nature of the Gaussian beam in which the power density is not evenly distributed across the beam spot (see Figure 15d).

**Figure 16:**
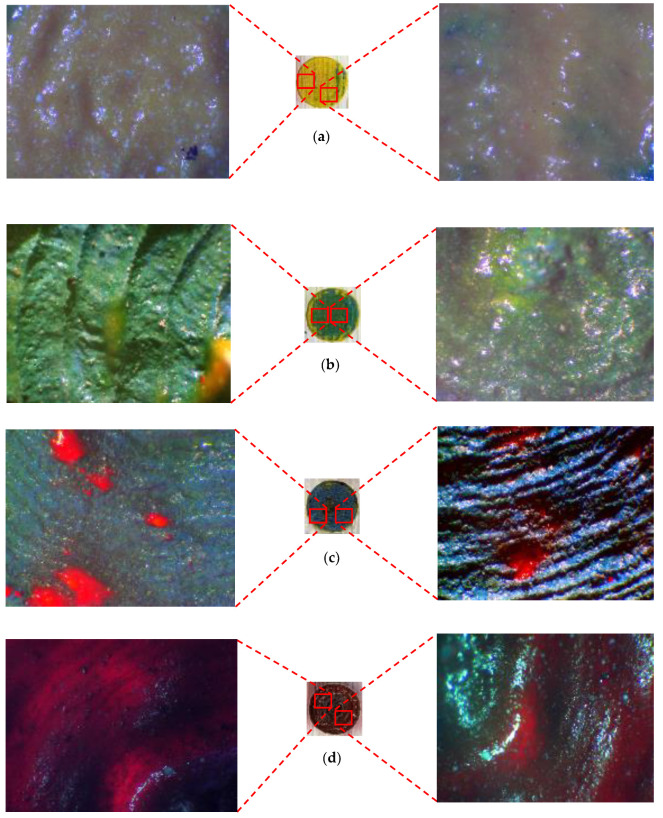
The final color design for optimum laser parameters: (**a**) yellow, (**b**) green, (**c**) blue, and (**d**) red-brown.

Figure 16 shows all finalized color designs using optimum laser parameters. Four colors were achieved, which were all used to create the final CAD design. Table 4 shows the exact parameters used in creating the final painting. It can be observed that blue color and red-brown color were achieved when multipass processing was utilized.

### Final CAD Design Micrographs

**Figure 17:**
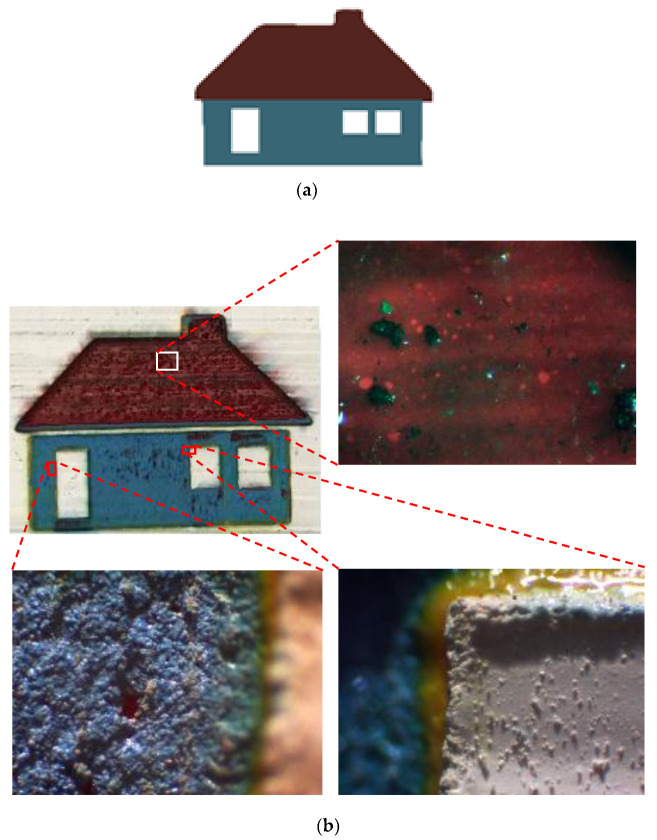
(**a**) CAD design of house. (**b**) Captured micrographs from different sections of laser-decolorized house.

**Figure 18:**
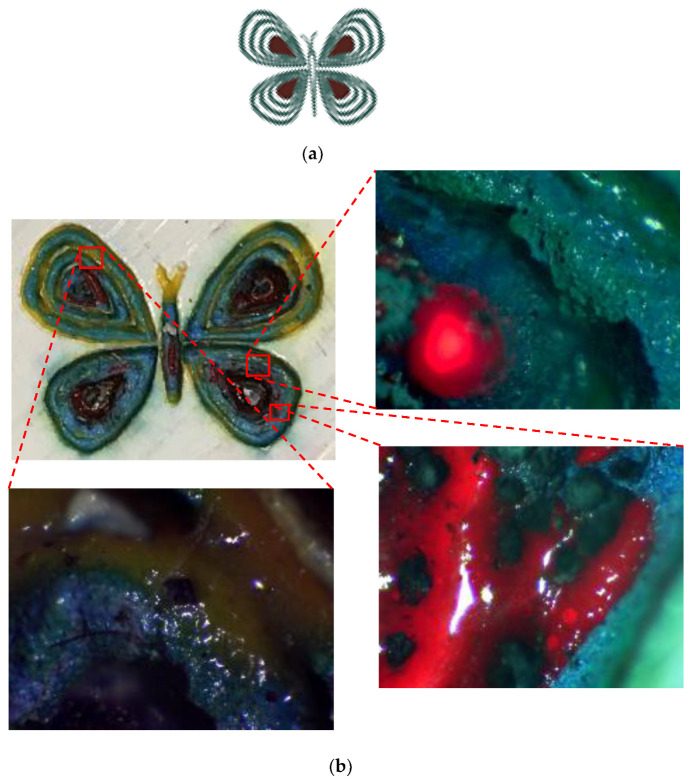
(**a**) CAD design of butterfly. (**b**) Captured micrographs from different sections of laser-decolorized butterfly.

**Figure 19:**
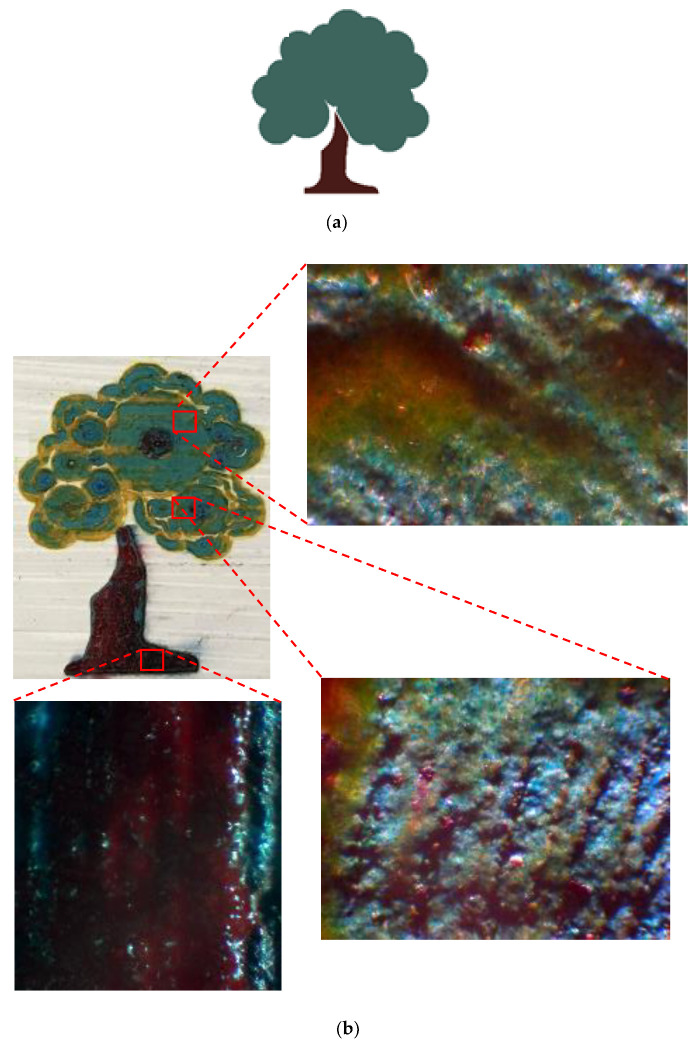
(**a**) CAD design of tree. (**b**) Captured micrographs from different sections of laser-decolorized tree.

Different parts of the final design were selected and, for each, micrographs were captured using a digital microscope. Great accuracy can be observed between the desired design and what was achieved by laser. Figure 17, Figure 18 and Figure 19 illustrate different areas selected for each pattern (house, butterfly, and tree) for taking micrographs and inspecting the true color for each section. As can be seen in Figure 17, the desired colors for the house should be red and blue. Accordingly, the same colors were observed after laser discoloration. This is evidenced by the micrographs, where the quality of decolorized surface and color tones are distinguishable. Similar micrograph results are observed for butterfly design and tree design in Figure 18 and Figure 19, respectively. These results show that a laser can act as a promising tool to make a painting with many details for each pattern of the painting.

**Figure 20:**
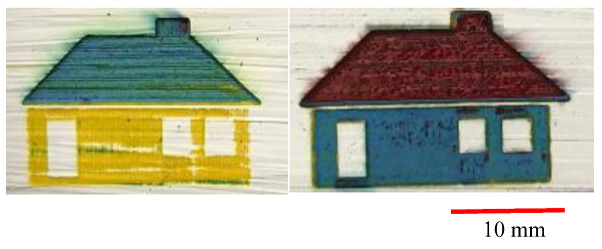
Different designs of house elements.

**Figure 21:**
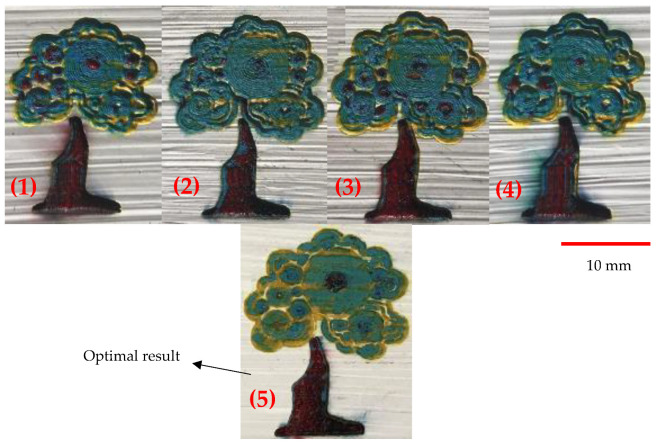
Different color designs of tree, 1–4: Different laser parameters (Table 3), 5: Optimum parameters (Table 4).

**Figure 22:**
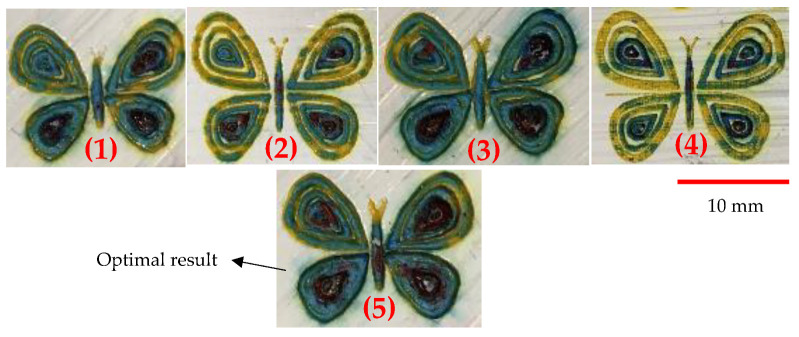
Different color designs of butterfly, 1–4: Different laser parameters (Table 3), 5: Optimum parameters (Table 4).

As shown previously, the final painting result includes a combination of natural elements (butterfly and tree) and simple construction design (house). Before the final painting, additional endeavors based on different laser parameters had been completed, which eventually resulted in different illustrations. For instance, for the “house” part, different colors of roof and body were obtained, shown in Figure 20. For the “tree” part, different trials of the laser decoloration method were completed, and each of them had different color designs, as shown in Figure 21. Additionally, some color designs of butterflies are illustrated in Figure 22.

## 5. Numerical Modelling

**Figure 23:**
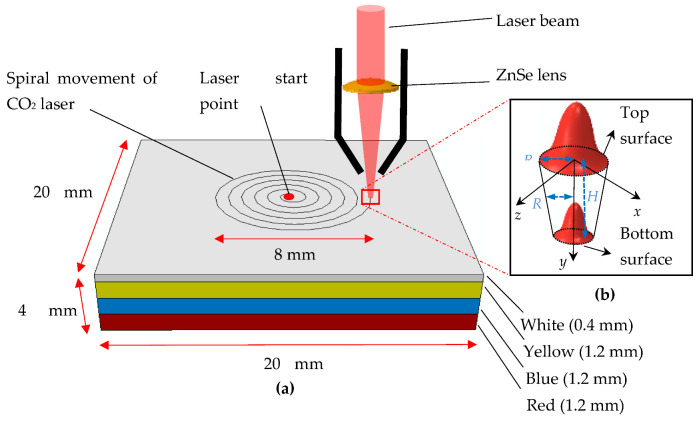
(**a**) Schematic diagram of simulated laser melting of colors. (**b**) 3D heat flux.

To simulate the flow mixing currents driven by thermal gradients, numerical simulations using the finite element method of laser processing were carried out. As the process involves temperature distribution and heat-affected zone (HAZ) at a fast pace, a detailed transient numerical simulation is required for resolving the physics. Figure 23a is a schematic of the simulation approach. In order to obtain each color using the laser processing parameters from Table 4, the laser source is defined as moving in a spiral movement. The thermal properties used for the simulations are presented in Table 5. The laser source model used for the thermal modeling and loads is

(1) $$Q=\frac{aP}{\pi R_{0}{}^{2}}\text{×}exp\left[-B{\left(\frac{R}{R_{0}}\right)}^{2}\right]$$

Here, the intensity of the laser is represented by *Q*, and the absorptivity is given by *a* and depends on the color (e.g., *a* ranges from 0.85 to 0.94 for yellow to red color). The laser power is represented by *P*, and the radius of the beam spot is represented by *R*_0_. The shape factor of the Gaussian distributed heat flux (schematically represented by Figure 23b) is given by *B*. The Abaqus software used for the simulation allowed the laser source modeling using DFLUX user subroutine as Gaussian volumetric heat flux [21], given by

(2) $$Q=\frac{aP}{{\pi R}^{2}H}\text{×}(1-\frac{y}{H})exp\left[-\left(\frac{{\left({z-z}_{t}\right)}^{2}+{\left({x-x}_{t}\right)}^{2}}{R_{0}{}^{2}}\right)\right]$$

where the substrate thickness is given by *H*, and the location of the spot is represented by *z_t_* and *x_t_*.

### 5.1. Geometric Model

**Figure 24:**
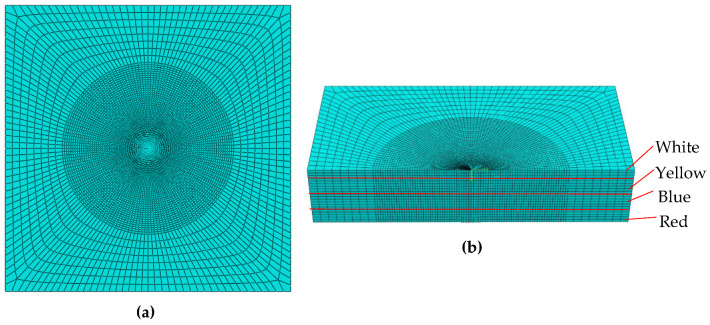
(**a**) Mesh distribution. (**b**) Isometric half view of denser mesh.

The geometrical model is a three-dimensional substrate undergoing a laser melting process. The substrate is of 20 × 20 × 4 size with dimensions in mm. The processing zone was discretized into 153,680 elements with 161,469 nodes. The element type is linear in nature with eight nodes (DCC3D8) allowing convection/diffusion for thermal analysis. As the trace of the laser source is spiral, the domain discretization was planned accordingly to introduce denser mesh along the path (Figure 24a), thus allowing temperature resolution along the path. Figure 24b shows the isometric cut section of the circular zone of melting involving denser mesh.

### 5.2. Thermal Analysis

The thermal analysis uses the traversing heat source along with the associated convective as well as radiative thermal boundary conditions. The spatial and temporally moving heat source representing moving CO_2_ laser [22,23] was used. The balance of energy on the substrate in this non-isothermal process is presented in mathematical form as follows:

(3) $$\rho \frac{{dC}_{p}T_{i}}{dt}=\nabla (K\nabla {T)+Q-Q}_{v}$$

where *ρ* is the density of the material (kg/m^3^), *K* is the thermal conductivity (W/mK), *C_p_* is the specific heat capacity (J/kgK), *T_i_* is the temperature (K), *t* is time variable (s), and *Q* is the heat source. The top surface of the acrylic paint sheet is involved convective and radiative heat loss flux, while the remaining surfaces are considered adiabatic. The corresponding convective flux *Q_v_* as the heat loss is given by

(4) $$Q_{v}=h\left(T_{s}{-T}_{0}\right){+\varepsilon }_{em}\sigma_{SBc}{(T}_{s}^{4}{-T}_{0}^{4})$$

where *h* is the heat transfer coefficient due to natural convection (= 30 W/m^2^K); *ε_em_* is the effective emissivity; *σ_SBc_* is the Stefan–Boltzmann constant (=5.67 × 10^−8^); and *T_s_* and *T*_0_ are the surface and the initial temperatures, respectively.

Other surfaces, including plate boundaries and edges, are considered adiabatic as follows:

(5) ∇(K∇T)=0

### 5.3. Numerical Result

**Figure 25:**
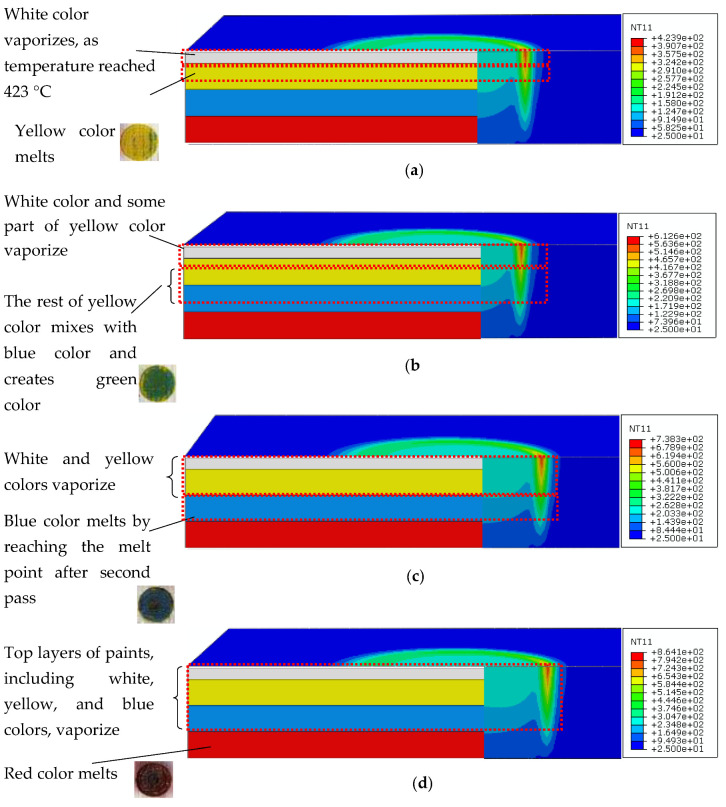
Temperature gradient for each color at last time increment: (**a**) yellow color, (**b**) green color, (**c**) blue color, and (**d**) red color.

Figure 25 represents the field temperature distributions for each color at the last time increment during the laser processing. It indicates that with the increase in laser speed, the temperature gradient is lowered significantly in the melting zone. Meanwhile, the maximum temperature can be observed at the top surface of the painted region, indicating white and yellow regions. It is pertinent to mention that after the melting and corresponding vaporization, the resultant yellow color is apparent on the paint surfaces (Figure 25a). Thus, with the lowering of laser speed, i.e., increase in exposure time to heat flux, the cooling effect of the paint is diminished. This thereby increases the top surface temperature and increases temperature through the thickness of the substrate. As a result, it can be observed in Figure 2 (using TGA, thermogravimetric analysis) that the entire surface turns white, with a yellow portion indicating the melted and vaporized part. Meanwhile, a mixture of green color indicates the melt pool comprising a mixture of yellow and blue colors (Figure 25b). For obtaining the blue color, the laser power needs to increase, which is attained through the reduction in laser source speed. However, the real challenge is that the increase in laser source power, either through lowering cutting speed or by increasing the source power, results in the creation of burnt spots on the surface of the workpiece (Figure 15d). To address the overburning, a multipass laser traversing technique was employed that obtained the blue and red colors. In this technique, the initial pass helps in vaporizing the top layers (i.e., generating green color), and the subsequent pass results in the target color being obtained. As observed in Figure 25c, at the end of the second pass, the temperature rises to more than 400 °C, the paints of white and yellow colors disappear, and the surface of the workpiece retains only blue color after melting as it solidifies. In Figure 25d, it can be seen that after the melting and vaporization of the top paint layers, only red color appears on the surface. Moreover, the subsequent passes of laser at reduced laser speed provide ample time for a rise in the temperature of the irradiated zone, resulting in a high thermal gradient with red-colored melt.

**Figure 26:**
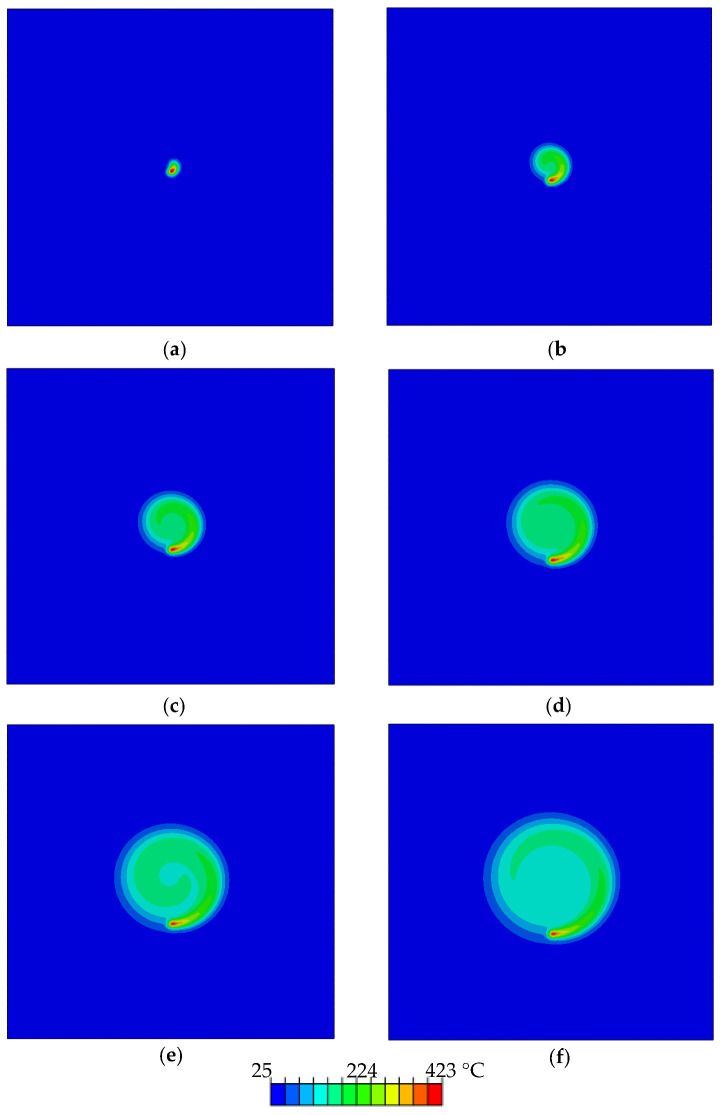
Temperature distribution at different time increments for melt pool of yellow color: (**a**) 0.14 s, (**b**) 0.85 s, (**c**) 2.14 s, (**d**) 3.9 s, (**e**) 6.42 s, and (**f**) 9.42 s.

Generally, the spiral motion of the beam allows the melted pool to cool down and lower the temperature in the initially melted zones, as evident by the nodal temperatures and the noted temperature gradients in NT11, Figure 26. The consequence is the provision of enough time for the solidification of the paints at the inner side of the melted pool through cooling. At the start of the process, the workpiece is kept at room temperature of 25 °C. The subsequent laser pass provides a sudden and sharp rise in the temperature of the neighborhood of the region. After the laser pass, rapid cooling takes place owing to convection as well as conductive losses, especially at the edges (Equation (4)). The gradient of the temperature in the core is driven by the heat absorption related to each paint, which is reduced along the direction of the thickness of the substrate (Figure 23b). The conical-shaped beam creates the patterns through heating, melting, and vaporizing as it moves, thereby generating different colors. In addition, the heat imparted is also absorbed as the shape of the heat flux (conical) creates high intensity at the center with an exponential drop in its periphery [24,25]. Consequently, this variation of the heat concentration within the beam also results in a significant gradient of the temperature along the direction of the depth of the specimen.

## 6. Conclusions

In this work, a continuous-wave CO_2_ laser was employed to create a method for painting artwork. Laser beam energy was applied as an image creation tool to provide graphic patterns and colored effects through a computer-aided design (CAD) approach. The use of optimal parameters allowed the creation of different colors for the provided drawing by CAD design. It was found that a colored surface with lower roughness results in better output colors as the absorbed laser energy is not overly reflected by the rugged surface, and it causes smoother melting/vaporizing of paints. By positioning the focal lens at an upper location and selecting the lowest laser power and minimum speed, a melt pool of colors was created, which could produce and mix high-quality tones of colors. The optimum laser processing parameters varied based on the sequence of colors positioned from top to bottom of the painted surface. For yellow and green colors, a single 0.4 W laser pass at 6.66 mm/s and 5 mm/s, respectively, was needed. On the contrary, for blue and red-brown colors, a multipass technique needed to be employed for primarily vaporizing the top layer of paints in the first pass and then starting to create a melt pool of the bottom paints in the second pass. The micrographs of the final painting demonstrate that the quality of decolorized surface and color tones are quite distinguishable and match exactly with what is needed based on CAD design. It was found that, unlike laser printers, using a laser to create artwork transfers the feeling of hand painting by creating real colors on the surface. In addition, the modeling and simulation results of the melting process indicate that the size of the melt pool decreases with the increase in the laser speed at a given power range and surface topology with prescribed thicknesses of the layered paints. Results indicate that the yellow color is seen at relatively higher laser speeds even with one pass. In order to obtain green color, the power of the laser has to be lowered especially through speed decrease. This provides sufficient time for yellow and blue colors to merge and melt. Meanwhile, the blue and red colors appear when two laser passes are employed, which ensures the removal of top layers of the paint without burnt regions. Moreover, the heat flux modeled as a conical volumetric heat source provides concentrated heat flux at the center of the top substrate surface, and thus more absorption is experienced at the top of the surface compared to the bottom side, where the temperature is significantly lower during the cutting process.

## Figures and Tables

**Table 1:** Illustrations and roughness measurements of different surfaces.

Surface	Roughness Measurement (Ra)	Sample Laser Discoloration
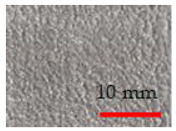	10.13	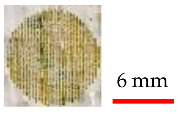
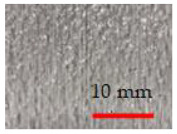	8.6	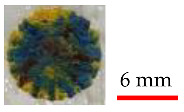
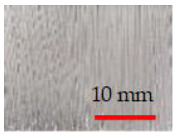	5.95	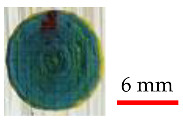

**Table 2:** Preliminary result of laser discoloration with different laser powers, speeds, and SODs.

Power (W)	Speed (mm/s)	SOD (mm)	No. of Passes	Color	Result
3.2	100	50.8	1	Yellow-blue-red	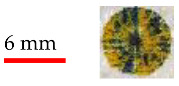
2	50	50.8	1	Blue-red-yellow	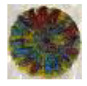
6	33.33	50.8	1	Blue-red-brown-yellow	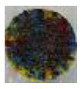
4.8	33.33	50.8	1	Blue-yellow	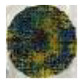
4	50	51	1	Burnt	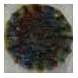
0.8	16.66	47.8	1	Burnt	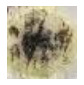
4	50	48.8	1	Burnt	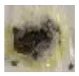
0.4	6.66	52	1	Yellow	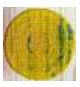
0.4	5	52	1	Green	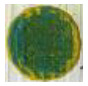
0.4	5	52	Pass 1	Blue	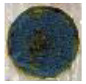
0.4	7.5	52	Pass 2		
0.4	5	52	Pass 1	Red-brown	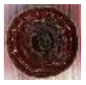
0.4	5.83	52	Pass 2		

**Table 3:** Preliminary results concerning different laser parameters and surface roughness values.

Set No.	Results	Ranges of Laser Parameters	Surface Roughness, Ra (μm)
1	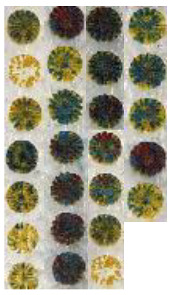	1 W ≤ P (laser power) ≤ 5 W 10 mm/s ≤ V (laser speed) ≤ 100 mm/s SOD = 50.8 mm	8.6
2	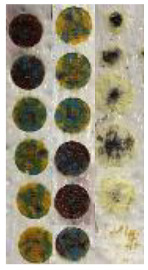	3 W ≤ P (laser power) ≤ 15 W 15 mm/s ≤ V (laser speed) ≤ 100 mm/s 47.8 mm ≤ SOD ≤ 51 mm	5.95
3	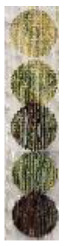	5 W ≤ P (laser power) ≤ 10 W 1 mm/s ≤ V (laser speed) ≤ 5 mm/s 49.8 mm ≤ SOD ≤ 50.8 mm	10.13
4	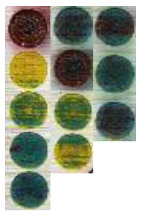	0.2 W ≤ P (laser power) ≤ 0.6 W 4 mm/s ≤ V (laser speed) ≤ 8 mm/s SOD = 52 mm (optimum results)	5.95

**Table 4:** Optimum laser processing parameters to obtain yellow, green, blue, and red-brown colors.

Power (W)	Speed (mm/s)	SOD (mm)	No. of Passes	Color	Result
0.4	6.66	52	1	Yellow	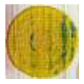
0.4	5	52	1	Green	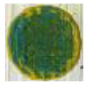
0.4	5	52	Pass 1	Blue	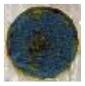
0.4	7.5	52	Pass 2		
0.4	5	52	Pass 1	Red-brown	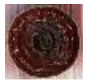
0.4	5.83	52	Pass 2		

**Table 5:** Thermal properties of paints [20].

**Acrylic Paints**	**Thermal Conductivity (W/mK)**	**Specific Heat (J/kgK)**	**Thermal Expansion (1/K)**
	1.45	5184	1.68 × 10^−4^

## Data Availability

Data is contained within the article.
